# Combined impact of prediabetes and fatty liver index on cardiometabolic outcomes and mortality in middle aged adults: a nationwide cohort study

**DOI:** 10.1186/s12933-025-02793-7

**Published:** 2025-07-10

**Authors:** Young Sang Lyu, Minae Park, Hee Kyung Kim, Sojeong Park, Ji Yong Park, A Ram Hong, Jee Hee Yoon, Seogsong Jeong, Youngmin Yoon, Jin Hwa Kim, Sang Yong Kim, Ho-Cheol Kang, Wonsuk Choi

**Affiliations:** 1 Division of Endocrinology and Metabolism, Department of Internal Medicine, Chosun University Hospital, Gwangju, Republic of Korea; 2 Data Science Team, Hanmi Pharm. Co., Ltd., Seoul, Republic of Korea; 3 Department of Internal Medicine, Chonnam National University Hwasun Hospital, Chonnam National University Medical School, 322, Seoyang-ro, Hwasun-eup, Hwasun-gun, Hwasun, 58128 Jeollanam-do Republic of Korea; 4 Department of Biomedical Informatics, Korea University College of Medicine, Seoul, Republic of Korea; 5 Division of Nephrology, Department of Medicine, Chosun University Hospital, Chosun University School of Medicine, Gwangju, Republic of Korea; 6 Department of Biological Chemistry, University of California Irvine School of Medicine, Irvine, CA USA

**Keywords:** Prediabetes, Hepatic steatosis, Middle-aged adult, Cardiometabolic disease, Cohort study

## Abstract

**Background:**

To investigate the combined effect of prediabetes and fatty liver index on incident diabetes (DM), major adverse cardiovascular events (MACE), and mortality in middle-aged adults.

**Methods:**

A nationwide cohort study was conducted involving 1,182,751 middle-aged adults aged 40 to 65 years, all of whom had no history of diabetes or cardiovascular disease. The primary outcomes of our study included incident DM, composite MACE and all-cause mortality.

**Results:**

Among the participants, 24.6% were diagnosed with prediabetes, while 8.8% had FLI ≥ 60 at baseline. Both conditions independently increased the risk of incident DM, composite MACE, and all-cause mortality. Stratification based on the presence of prediabetes and FLI ≥ 60 showed that their combination posed the highest risk for outcomes, even after adjusting for relevant covariates. For incident DM, the odds ratios (ORs) with 95% confidence intervals (CI) were as follows: 3.75 (3.69–3.81), 2.35 (2.29–2.42), and 6.80 (6.62–6.98) for prediabetes with FLI < 60, normoglycemia with FLI ≥ 60, and prediabetes with FLI ≥ 60, respectively. For composite MACE, the ORs (95% CI) were 1.02 (1.00–1.05), 1.23 (1.17–1.28), and 1.27 (1.21–1.33) for prediabetes with FLI < 60, normoglycemia with FLI ≥ 60, and prediabetes with FLI ≥ 60, respectively. For all-cause mortality, ORs (95% CI) were 1.12 (1.08–1.15), 1.51 (1.43–1.59), and 1.69 (1.60–1.79) for prediabetes with FLI < 60, normoglycemia with FLI ≥ 60, and prediabetes with FLI ≥ 60, respectively.

**Conclusion:**

The coexistence of prediabetes and FLI ≥ 60, which is a surrogate marker of hepatic steatosis, demonstrated a combined effect, additively increasing the risk of incident DM, composite MACE, and all-cause mortality in middle-aged adults.

**Trial registration:**

Not applicable (retrospectively registered).

**Graphical abstract:**

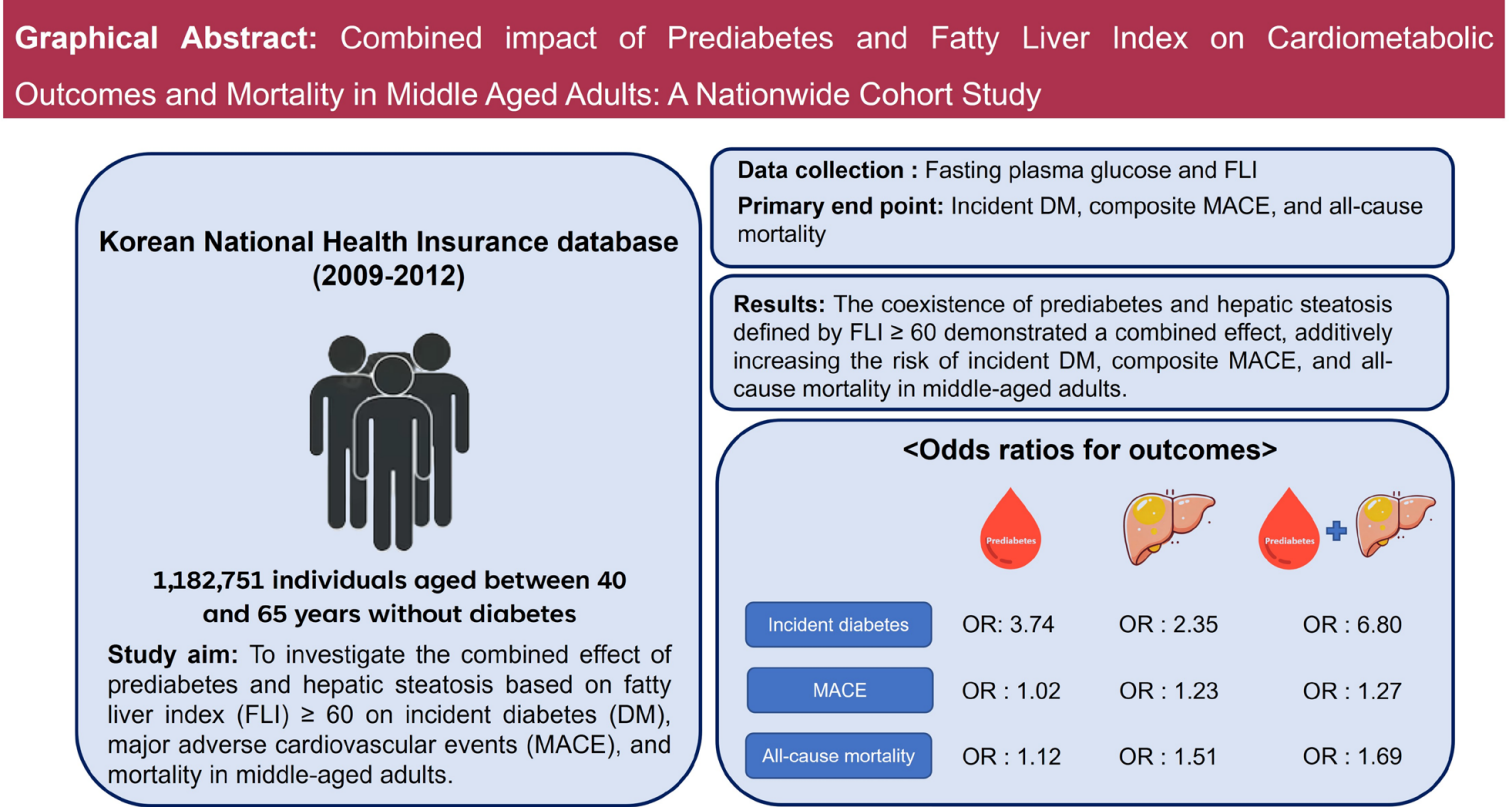

**Supplementary Information:**

The online version contains supplementary material available at 10.1186/s12933-025-02793-7.

## Research insights

**What is currently known about this topic?**

- Prediabetes and hepatic steatosis are increasingly prevalent conditions that contribute to the incidence of type 2 diabetes (DM) in middle-aged adults.
- The impact of prediabetes on major adverse cardiovascular events (MACE) is controversial and may vary depending on age and ethnicity, while the association between hepatic steatosis and MACE is consistently reported in middle-aged adults.
- The effects of prediabetes and hepatic steatosis on mortality have been reported in middle-aged adults, but these studies are limited by factors such as small sample sizes and insufficient adjustment for confounders.

**What is the key research question?**

- Does the coexistence of prediabetes and hepatic steatosis based on FLI ≥ 60 increase the risk of incident DM, MACE, and all-cause mortality in middle-aged adults?

**What is new?**

- Prediabetes and FLI ≥ 60 independently increase the risk of incident DM, composite MACE, and all-cause mortality in middle-aged adults.
- The coexistence of both conditions additively contributes to the increased risk of incident DM, composite MACE, and all-cause mortality in middle-aged adults.

**How might this study influence clinical practice?**

- Screening for hepatic steatosis based on FLI ≥ 60 in individuals with prediabetes may help identify high-risk individuals and enable early interventions to reduce cardiometabolic complications and mortality.

## Background

Prediabetes and hepatic steatosis are increasingly prevalent conditions that pose significant global health risks. Prediabetes is characterized by elevated blood glucose levels that have not yet reached the threshold for a diabetes diagnosis, affecting a substantial portion of the adult population [1]. Similarly, hepatic steatosis, characterized by the accumulation of fat in the liver, has emerged as one of the most common liver disorders globally [2, 3]. Both conditions are associated with various metabolic abnormalities and play a significant role in the global burden of chronic diseases. These conditions contribute to the increased prevalence of type 2 diabetes (DM), cardiovascular disease (CVD), and other health-related complications [4–7].

Numerous studies have highlighted the complex, bidirectional relationship between prediabetes and hepatic steatosis, both of which share the common underlying mechanism of insulin resistance (IR). Furthermore, 38–75% of patients with prediabetes are estimated to have hepatic steatosis [8, 9]. This combination may synergistically exacerbate cardiometabolic outcomes, leading to the development of DM and CVD and, eventually, mortality. While several studies have linked DM and hepatic steatosis to an increased risk of CVD and mortality [10–13], research focused specifically on prediabetes and hepatic steatosis is limited.

Therefore, this study aimed to investigate the combined effects of prediabetes and hepatic steatosis defined by fatty liver index (FLI) on cardiometabolic outcomes and all-cause mortality in middle-aged adults, using a nationwide cohort from Korea.

## Methods

### Data source

The Korean National Health Insurance (NHIS) datasets, which cover claims and preventive health check-ups in Korea from January 2009 to December 2012, were employed in this study. The NHIS, a single-payer healthcare system administered by the Korean government, insures > 97% of the Korean population. This database includes medical diagnoses classified by International Classification of Diseases 10th revision (ICD-10) codes, alongside comprehensive details regarding prescriptions, procedures, hospital visits, and hospitalizations [14]. The health check-up dataset comprises questionnaires on lifestyle and behaviors, along with anthropometric and laboratory measurements. The methodology for these variables has been detailed in previous studies [15, 16]. Additionally, we utilized nationwide death certificate data provided by the Korean National Statistical Office.

### Study cohort

We selected individuals aged between 40 and 65 years without diabetes—defined as fasting glucose levels of ≥ 126 mg/dL, presence of ICD-10 codes E10-14, or a history of glucose-lowering medication before the index date—and who had undergone routine health check-ups that included FLI calculation data from January 2009 to December 2012. Of 3,602,623 participants, 2,419,872 individuals were excluded due to factors such as age (younger than 40 years old or ≥ 65 years old), cardiovascular (ICD-10 codes I21–22, I48, and I63–64) or liver disease other than nonalcoholic fatty liver disease (ICD-10 codes K75.1–75.4, K75.9, and K76.1–76.9), alcohol consumption ≥ 3 times per week, any cancer diagnosis (ICD-10 codes C00–97), rheumatic mitral valve disease (ICD-10 codes I05), cardiac or vascular implants or grafts (ICD-10 code Z95), and missing data. Overall, 1,182,751 participants were included in this study (Supplementary Fig. 1), with the index date defined as the first-day FLI calculation data collected.

### Definition of prediabetes

Prediabetes was defined as a fasting glucose level ranging from 100 to 125 mg/dL in individuals without a history of diabetes. A fasting glucose < 100 mg/dL was considered indicative of normoglycemia in the same population.

### Estimation of hepatic steatosis based on FLI

Hepatic steatosis was identified using the FLI, with a threshold set at ≥ 60. This index is a validated, noninvasive method for diagnosing hepatic steatosis, and it has demonstrated significant accuracy and reliability, particularly in Asian populations [17]. The FLI was calculated using this following formula: (e^0.953×ln(TG)+0.139×body mass index+0.718×ln(GGT)+0.053×waist circumference−15.745^) ÷ (1 + e^0.953×ln(TG)+0.139×body mass index+0.718×ln(GGT)+0.053×waist circumference−15.745^)×100, where TG = triglyceride and GGT = γ-glutamyl transferase [18].

### Study endpoint and follow-up

The outcomes of this study included incident DM, composite major adverse cardiovascular events (MACE)—including myocardial infarction (MI), stroke, or cardiovascular death—and all-cause mortality. Incident DM was defined as a new DM diagnosis (ICD-10 codes E10-14) accompanied by a prescription for glucose-lowering medications. MI was defined as hospitalization with ICD-10 codes I21 or I22. Stroke was defined as hospitalization with ICD-10 codes I63 or I64, accompanied by claims for brain imaging, such as MRI or CT. Cardiovascular death was identified using ICD-10 codes I00–I99. The study population was followed from the index date until the occurrence of each study outcome, death, or the end of the study period on December 31, 2021.

### Definitions of variables

Data on current smoking, alcohol consumption, and regular exercise were collected through surveys. Mild alcohol consumption was defined as drinking fewer than three times per week. Regular physical activity was classified as high-intensity activity (causing extreme breathlessness) performed at least three times per week or moderate-intensity activity (causing significant breathlessness) performed at least five times per week. Income was classified into quartiles based on monthly income, with particular emphasis on the lowest quartile. Body mass index (BMI) was computed by dividing the weight of the participants in kilograms by their height in meters squared. Glucose and lipid levels were measured after an overnight fast. Hypertension (HTN) was defined by meeting one or more of the following criteria: specific ICD-10 codes, prescription of antihypertensive medication, or a systolic blood pressure (BP) of ≥ 140 mmHg or diastolic BP of ≥ 90 mmHg during the baseline assessment. Dyslipidemia was defined by meeting one or more of the following criteria: specific ICD-10 codes, prescription of lipid-lowering medication, or a total cholesterol level of ≥ 240 mg/dL during the baseline assessment. Chronic kidney disease (CKD) was defined as meeting criteria such as one claim per year with ICD-10 codes N18 or N19.

### Statistical analysis

Data are reported as means and standard deviation for continuous variables and as counts (with percentages) for categorical variables. For comparisons between two groups, Student’s t-test was used for normally distributed continuous variables, while the Wilcoxon rank-sum test was applied for non-normally distributed continuous variables. Categorical variables were compared using the Chi-square test. For comparisons among four groups, ANOVA (Analysis of Variance) was used for normally distributed continuous variables, whereas the Kruskal-Wallis H test was applied for non-normally distributed continuous variables. Kaplan–Meier curves were used to illustrate the cumulative incidence of incident DM, composite MACE, and all-cause mortality. Differences between groups were evaluated using the log-rank test. Cox regression analyses were conducted to provide hazard ratios and 95% confidence intervals (CIs) for the rates of these outcomes. Since some outcome analyses did not meet the proportional hazards assumption, we also conducted logistic regression analyses to provide odds ratios (ORs) and 95% CIs for the outcome rates. For analyses adjusted for multiple variables, model 1 included adjustments for age and sex. Model 2 was further adjusted for income, smoking status, alcohol consumption, regular exercise, body weight, HTN, dyslipidemia, and chronic kidney disease (CKD). A *P*-value of < 0.05 was considered statistically significant across all tests. Statistical procedures were conducted using SAS version 9.4 (SAS Institute, Cary, NC, USA).

## Results

### Baseline characteristics

Table 1 shows the baseline characteristics of the study participants categorized based on prediabetes and FLI ≥ 60. The study included 1,182,751 middle-aged adults, among whom 290,726 (24.6%) were diagnosed with prediabetes and 103,364 (8.7%) had FLI ≥ 60. Baseline characteristics revealed significant differences between the groups based on prediabetes and FLI ≥ 60. Participants with prediabetes were more likely to be older and male, and they had higher risks of current smoking and alcohol consumption. They also had greater body weight, BMI, waist circumference, blood pressure, fasting glucose, liver enzyme, and lipid levels. Additionally, they exhibited a higher risk of comorbidities such as hypertension, dyslipidemia, and CKD. Participants with FLI ≥ 60 exhibited a similar pattern, except for engaging in less regular physical activity.

**Table 1:** Baseline characteristics of the study participants based on prediabetes and FLI status

	Prediabetes status			FLI status		
	Normoglycemia (75.4%, *n* = 892,025)	Prediabetes (24.6%, *n* = 290,726)	*P*-value	FLI < 60 (91.2%, *n* = 1,079,387)	FLI ≥ 60 (8.8%, *n* = 103,364)	*P*-value
Age, years	48.6 ± 6.7	49.7 ± 6.8	< 0.001	48.9 ± 6.8	48.6 ± 6.7	< 0.001
Men	337,557 (37.8)	147,142 (50.6)	< 0.001	402,970 (37.3)	81,729 (79.1)	< 0.001
Income level, lowest 25%	184,613 (20.7)	58,591 (20.2)	< 0.001	225,001 (20.9)	18,203 (17.6)	< 0.001
Smoking			< 0.001			< 0.001
Nonsmoker	636,640 (71.4)	183,163 (63.0)		778,061 (72.1)	41,742 (40.4)	
Former smoker	97,145 (10.9)	44,075 (15.2)		118,930 (11.0)	22,290 (21.6)	
Current smoker	158,240 (17.7)	63,488 (21.8)		182,396 (16.9)	39,332 (38.1)	
Alcohol			< 0.001			< 0.001
None	576,875 (64.7)	167,653 (57.7)		701,037 (65.0)	43,491 (42.1)	
Mild	315,150 (35.3)	123,073 (42.3)		378,350 (35.1)	59,873 (57.9)	
Regular physical activity	157,588 (17.7)	52,467 (18.1)	< 0.001	193,918 (18.0)	16,137 (15.6)	< 0.001
Body weight, kg	61.3 ± 10.2	64.8 ± 10.8	< 0.001	60.7 ± 9.2	77.9 ± 9.6	< 0.001
BMI, kg/m^2^	23.4 ± 2.9	24.3 ± 3.1	< 0.001	23.2 ± 2.6	27.9 ± 3.0	< 0.001
BMI			< 0.001			< 0.001
< 18.5 kg/m^2^	24,552 (2.8)	4633 (1.6)		29,161 (2.7)	24 (0.0)	
18.5–22.9 kg/m^2^	393,812 (44.2)	93,632 (32.2)		485,041 (44.9)	2403 (2.3)	
23.0–24.9 kg/m^2^	229,469 (25.7)	78,338 (27.0)		296,589 (27.5)	11,218 (10.9)	
25.0–29.9 kg/m^2^	223,892 (25.1)	101,890 (35.1)		257,986 (23.9)	67,796 (65.6)	
≥ 30.0 kg/m^2^	20,300 (2.3)	12,233 (4.2)		10,610 (1.0)	21,923 (21.2)	
Waist circumference						
In men	82.9 ± 7.3	84.7 ± 7.3	< 0.001	81.8 ± 6.4	91.7 ± 6.2	< 0.001
In women	75.3 ± 7.7	77.9 ± 8.3	< 0.001	75.3 ± 7.3	92.4 ± 7.5	< 0.001
SBP, mmHg	119.6 ± 14.5	124.6 ± 15.2	< 0.001	120.0 ± 14.6	129.3 ± 14.9	< 0.001
DBP, mmHg	74.9 ± 10.1	78.1 ± 10.4	< 0.001	75.1 ± 10.0	81.8 ± 10.4	< 0.001
Fasting glucose, mg/dL	88.2 ± 7.4	107.4 ± 6.4	< 0.001	92.5 ± 10.7	97.7 ± 11.9	< 0.001
Total cholesterol, mg/dL	197.6 ± 37.9	206.3 ± 42.1	< 0.001	198.2 ± 38.5	216.0 ± 41.9	< 0.001
Triglycerides, mg/dL	119.0 ± 84.1	143.6 ± 99.6	< 0.001	112.5 ± 64.4	256.6 ± 167.2	< 0.001
HDL-C, mg/dL	57.0 ± 27.8	55.4 ± 31.9	< 0.001	57.1 ± 27.2	50.9 ± 42.3	< 0.001
LDL-C, mg/dL	119.6 ± 66.9	124.6 ± 70.8	< 0.001	120.7 ± 63.9	122.4 ± 100.4	< 0.001
AST, IU/L^a^	23 (19–28)	22 (18–26)	< 0.001	28 (23–35)	21 (18–26)	< 0.001
ALT, IU/L^a^	21 (16–30)	18 (14–25)	< 0.001	34 (25–49)	18 (14–25)	< 0.001
GGT, IU/L^a^	25 (17–41)	19 (14–30)	< 0.001	56 (37–90)	19 (14–29)	< 0.001
Hypertension	155,818 (17.5)	76,582 (26.3)	< 0.001	194,874 (18.1)	37,526 (36.3)	< 0.001
Dyslipidemia	154,595 (17.3)	69,839 (24.0)	< 0.001	189,934 (17.6)	34,500 (33.4)	< 0.001
Chronic kidney disease	568 (0.1)	194 (0.1)	0.573	683 (0.1)	79 (0.1)	0.114

Continuous variables are expressed as mean ± standard deviation. Categorical data are presented as frequencies and percentages

FLI, fatty liver index; BMI, body mass index; SBP, systolic blood pressure; DBP, diastolic blood pressure; AST, aspartate aminotransferase; ALT, alanine aminotransferase; GGT, gamma-glutamyl transferase

^a^As the variables did not follow a normal distribution, they are presented as median (interquartile range), and group comparisons were conducted using the Wilcoxon rank sum test

### Follow-up duration and incidence of outcomes

The median follow-up duration was 11.68 years (interquartile range [IQR], 1.47), which was similar across different prediabetes statuses: 11.67 (IQR, 1.47) and 11.72 years (IQR, 1.45) for normoglycemia and prediabetes, respectively. Similarly, it was comparable across FLI statuses: 11.68 (IQR, 1.46) and 11.75 years (IQR, 1.51) for individuals with FLI < 60 and FLI ≥ 60, respectively. During follow-up, 94,350 (7.9%), 35,908 (3.0%), and 25,130 (2.1%) cases of incident DM, composite MACE, and all-cause mortality, respectively, were observed.

### Risk of outcomes based on prediabetes status in middle-aged adults

Event-free survival for cardiometabolic outcomes and all-cause mortality, as shown in Kaplan–Meier curves (Supplementary Fig. 2), indicated a significantly higher cumulative incidence of all outcomes among participants with prediabetes. Participants with prediabetes had a significantly increased risk of incident DM, composite MACE, and all-cause mortality, even after adjusting for relevant covariates in Model 2 (Table 2).

**Table 2:** Incidence rates and risk of outcomes based on prediabetes status in middle-aged adults

	Event	Duration (person-years)	Incidence Rate^a^	Hazard Ratio				Odds ratio			
				Model 1 (95% CI)	*P*-value	Model 2 (95% CI)	*P*-value	Model 1 (95% CI)	*P*-value	Model 2 (95% CI)	*P*-value
Incident diabetes											
Normoglycemia	42,553	9,995,192	4.26	Reference		Reference		Reference		Reference	
Prediabetes	51,797	3,058,358	16.94	3.81 (3.76–3.86)	< 0.001	3.34 (3.29–3.38)	< 0.001	4.06 (4.00-4.11)	< 0.001	3.61 (3.56–3.66)	< 0.001
Composite MACE											
Normoglycemia	25,515	10,047,591	2.54	Reference		Reference		Reference		Reference	
Prediabetes	10,393	3,258,643	3.19	1.09 (1.06–1.11)	< 0.001	1.04 (1.02–1.06)	0.001	1.09 (1.06–1.11)	< 0.001	1.03 (1.01–1.06)	0.006
All-cause mortality											
Normoglycemia	17,418	10,152,104	1.72	Reference		Reference		Reference		Reference	
Prediabetes	7,712	3,301,431	2.34	1.13 (1.10–1.16)	< 0.001	1.14 (1.11–1.17)	< 0.001	1.13 (1.09–1.16)	< 0.001	1.14 (1.10–1.17)	< 0.001

CI, confidence interval; MACE, major adverse cardiovascular events

^a^Incidence for 1000 person-years. Model 1: Adjusted for age and sex. Model 2: Adjusted for age, sex, income, smoking status, alcohol consumption, regular physical activity, body weight, hypertension, dyslipidemia, and chronic kidney disease

### Risk of outcomes based on FLI ≥ 60 in middle-aged adults

Event-free survival for cardiometabolic outcomes and all-cause mortality, as shown in Kaplan–Meier curves (Supplementary Fig. 3), revealed a significantly higher cumulative incidence of all outcomes among participants with FLI ≥ 60. Participants with FLI ≥ 60 had a significantly increased risk of incident DM, composite MACE, and all-cause mortality, even after adjusting for relevant covariates in Model 2 (Table 3).

**Table 3:** Incidence rates and risk of outcomes based on FLI in middle-aged adults

	Event	Duration (person-years)	Incidence Rate^a^	Hazard Ratio				Odds Ratio			
				Model 1 (95% CI)	*P*-value	Model 2 (95% CI)	*P*-value	Model 1 (95% CI)	*P*-value	Model 2 (95% CI)	*P*-value
Incident diabetes											
FLI < 60	70,059	11,995,059	5.84	Reference		Reference		Reference		Reference	
FLI ≥ 60	24,291	1,058,492	22.95	3.99 (3.93–4.06)	< 0.001	2.06 (2.02–2.10)	< 0.001	4.41 (4.33–4.49)	< 0.001	2.20 (2.15–2.24)	< 0.001
Composite MACE											
FLI < 60	30,897	12,156,577	2.54	NA	NA	NA	NA	Reference		Reference	
FLI ≥ 60	5,011	1,149,657	4.36					1.46 (1.41–1.51)	< 0.001	1.23 (1.19–1.28)	0.006
All-cause mortality											
FLI < 60	21,612	12,282,874	1.76	Reference		Reference		Reference		Reference	
FLI ≥ 60	3,518	1,170,661	3.01	1.34 (1.29–1.39)	< 0.001	1.51 (1.45–1.58)	< 0.001	1.34 (1.29–1.39)	< 0.001	1.53 (1.46–1.59)	< 0.001

CI, confidence interval; FLI, fatty liver index; MACE, major adverse cardiovascular events; NA, not applicable

^a^Incidence for 1000 person-years. Model 1: Adjusted for age and sex. Model 2: Adjusted for age, sex, income, smoking status, alcohol consumption, regular physical activity, body weight, hypertension, dyslipidemia, and chronic kidney disease

### Risk of outcomes based on prediabetes and FLI ≥ 60 in middle-aged adults

**Fig. 1:**
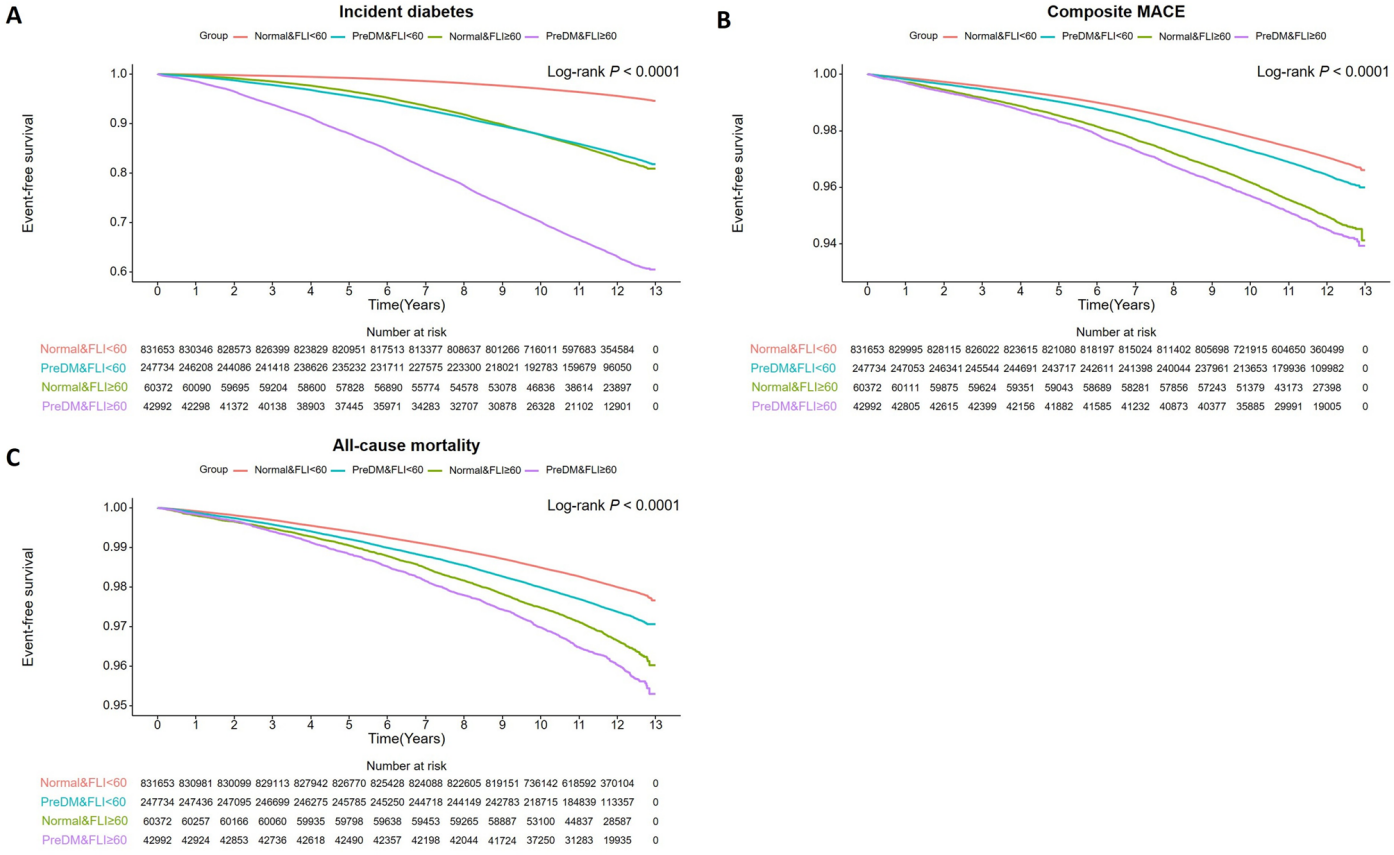
Kaplan–Meier estimates of outcomes based on prediabetes and hepatic steatosis status in middle-aged adults. **A** Incident diabetes, **B** composite major adverse cardiovascular events, **C** all-cause mortality

Study participants were stratified based on prediabetes and FLI (Supplementary Table 1). Among 892,025 and 290,726 individuals with normoglycemia and prediabetes, 60,372 (6.7%) and 42,992 (14.7%) had FLI ≥ 60, respectively. Event-free survival for incident DM, composite MACE, and all-cause mortality, based on prediabetes and FLI ≥ 60 status, is depicted in Kaplan–Meier curves (Fig. 1). The curves showed that the highest cumulative incidence of all outcomes occurred in the prediabetes group with FLI ≥ 60, followed by the normoglycemia group with FLI ≥ 60, prediabetes group with FLI < 60, and normoglycemia with FLI < 60. Table 4 presents the ORs for incident DM, composite MACE, and all-cause mortality based on prediabetes and FLI status. After adjusting for relevant factors (Model 2), the normoglycemia group with FLI ≥ 60 had a higher risk for all three outcomes than those of the normoglycemia group with FLI < 60. The presence of prediabetes and FLI ≥ 60 significantly amplified the risk for all three outcomes, even after adjusting for relevant covariates (Model 2). Additionally, the combined presence of prediabetes and FLI ≥ 60 demonstrated an additive increase in the risk of all outcomes (*P*_*trend*_ <0.001).

**Table 4:** Incidence rates and risk of outcomes based on prediabetes and FLI in middle-aged adults

	Event	Duration (person-years)	Incidence Rate^a^	Hazard Ratio				Odds Ratio			
				Model 1 (95% CI)	*P*-value	Model 2 (95% CI)	*P*-value	Model 1 (95% CI)	*P*-value	Model 2 (95% CI)	*P*-value
Incident diabetes											
Normoglycemia & FLI < 60	33,122	9,347,585	3.54	NA	NA	NA	NA	Reference		Reference	
Prediabetes & FLI < 60	36,937	2,647,474	13.95					4.05 (3.98–4.11)	< 0.001	3.75 (3.69–3.81)	< 0.001
Normoglycemia & FLI ≥ 60	9,431	9,347,585	14.56					4.60 (4.49–4.72)	< 0.001	2.35 (2.29–2.42)	< 0.001
Prediabetes & FLI ≥ 60	14,860	410,885	36.17					13.01 (12.71–13.33)	< 0.001	6.80 (6.62–6.98)	< 0.001
*P*_trend_									< 0.001		< 0.001
Composite MACE											
Normoglycemia & FLI < 60	22,693	9,373,759	2.42	NA	NA	NA	NA	Reference		Reference	
Prediabetes & FLI < 60	8,204	2,782,818	2.95					1.06 (1.03–1.09)	< 0.001	1.02 (1.00-1.05)	0.070
Normoglycemia & FLI ≥ 60	2,822	673,832	4.19					1.46 (1.40–1.52)	< 0.001	1.23 (1.17–1.28)	< 0.001
Prediabetes & FLI ≥ 60	2,189	475,825	4.60					1.52 (1.45–1.59)	< 0.001	1.27 (1.21–1.33)	< 0.001
*P*_trend_									< 0.001		< 0.001
All-cause mortality											
Normoglycemia & FLI < 60	15,509	9,466,634	1.64	NA	NA	NA	NA	Reference		Reference	
Prediabetes & FLI < 60	6,103	2,816,240	2.17					1.10 (1.07–1.13)	< 0.001	1.12 (1.08–1.15)	< 0.001
Normoglycemia & FLI ≥ 60	1,909	685,470	2.78					1.32 (1.25–1.38)	< 0.001	1.51 (1.43–1.59)	< 0.001
Prediabetes & FLI ≥ 60	1,609	485,191	3.32					1.47 (1.39–1.55)	< 0.001	1.69 (1.60–1.79)	< 0.001
*P*_trend_									< 0.001		< 0.001

CI, confidence interval; MACE, major adverse cardiovascular events; NA, not applicable

^a^Incidence for 1000 person-years. Model 1: Adjusted for age and sex. Model 2: Adjusted for age, sex, income, smoking status, alcohol consumption, regular physical activity, body weight, hypertension, dyslipidemia, and chronic kidney disease

### Subgroup and sensitivity analyses

Subgroup analysis was conducted on incident DM, composite MACE, and all-cause mortality by stratifying participants based on baseline variables, including sex, income level, smoking status, alcohol consumption, and BMI value of 25 (Supplementary Tables 2–6). The combined effect of prediabetes and FLI ≥ 60 on all three outcomes was observed in both groups, with a stronger effect in the female, low income, non-current smoker, mild alcohol-consuming, and BMI < 25 kg/m^2^ groups.

For the sensitivity analysis, hepatic steatosis was defined as FLI ≥ 30 (Supplementary Table 7). The combined effect of prediabetes and FLI ≥ 30 on incident DM, composite MACE, and all-cause mortality was similar to the main analysis (Supplementary Table 8). Additionally, we further analyzed the 5-year and 10-year outcomes based on prediabetes and FLI status (Supplementary Tables 9 and 10). The combined effect of prediabetes and FLI ≥ 60 on all three outcomes was observed at both time points, although the effect diminished over time.

## Discussion

In this study, prediabetes and hepatic steatosis defined as FLI ≥ 60 were independently associated with an increased risk of incident DM, composite MACE, and all-cause mortality in middle-aged adults, even after adjusting for relevant covariates. The coexistence of both conditions was associated with an approximately 6.8-, 1.3-, and 1.7-fold increase in the risk of incident DM, composite MACE, and all-cause mortality, respectively. Moreover, each condition additively contributed to the increased risk of cardiometabolic outcomes and all-cause mortality.

Type 2 DM is a critical disease that can lead to complications and significantly reduce lifespan, making proactive management essential to prevent its onset [19, 20]. Studies show that prediabetes [21] and hepatic steatosis [22, 23] independently increase the risk of developing incident DM in middle-aged adults. In addition, one meta-analysis reported that hepatic steatosis is bidirectionally associated with the incidence of DM [24]. Although strong evidence indicates that both conditions individually elevate the risk of DM, it remains unclear whether their coexistence leads to a higher risk than either condition alone. Our study revealed that both conditions independently increased the risk of incident DM, which is consistent with previous research findings. Furthermore, we observed a significant combined effect when both conditions coexist, resulting in a substantially higher risk of DM than that associated with either condition alone. The Diabetes Prevention Program Outcomes [25] and the Framingham Offspring Studies [26] report that a significant proportion of individuals with prediabetes do not progress to diabetes, even after long-term follow-up. Therefore, identifying individuals with prediabetes at high risk of progressing to diabetes is crucial. Our findings suggest that assessing hepatic steatosis in individuals with prediabetes could be crucial in identifying those at higher risk of developing DM. This approach may enhance the effectiveness of screening and prevention strategies for incident DM in high-risk middle-aged adults.

Despite the high prevalence of prediabetes and hepatic steatosis, their association with CVD remains a topic of ongoing debate. Some research suggests that prediabetes independently contributes to the risk of MACE [27–29], while others report no such association [30, 31]. This discrepancy may be influenced by the presence of other risk factors such as obesity, hypertension, and dyslipidemia. Unlike the controversy surrounding prediabetes, hepatic steatosis consistently increases cardiovascular risk. Most studies show that hepatic steatosis, whether diagnosed radiologically [5, 6, 12] or defined as an FLI ≥ 60 [32, 33], is associated with an increased cardiovascular risk in the general population. Our study demonstrated that adults with prediabetes or hepatic steatosis based on FLI ≥ 60 face a higher risk of composite MACE compared to those without these conditions. Additionally, the risk is significantly increased when both risk factors are present simultaneously. Our study showed differing results regarding the association between prediabetes and CVD risk compared to those of certain previous studies [30, 31]. These differences may be attributed to factors such as the sample size, follow-up duration, and ethnicity of the study population. Given that our study involved a larger sample size and longer follow-up period, it likely provides data that more accurately reflects real-world conditions. Although the increase in the risk of MACE with prediabetes alone is relatively modest, the presence of hepatic steatosis in individuals with prediabetes significantly elevates the OR. This offers valuable insights for identifying individuals at high risk for CVD.

Evidence regarding the effect of prediabetes and hepatic steatosis on mortality in middle-aged adults is limited. Some studies indicate that prediabetes [34, 35] and hepatic steatosis [36] independently increase the risk of mortality in this population. However, these studies are limited by factors such as relatively small sample sizes, insufficient adjustment for confounders, or potential misclassification bias in death certificate data. In our large-scale cohort study, even after sufficiently adjusting for risk variables, participants with prediabetes or hepatic steatosis had a higher all-cause mortality rate in middle-aged adults. Furthermore, when both conditions were present simultaneously, the mortality rate increased even further. This finding highlights the compounded risk these conditions posed by the coexisting of these conditions, emphasizing the importance of their early detection and management to mitigate mortality rates.

The exact mechanism underlying the synergistic detrimental effects of prediabetes and hepatic steatosis remains unclear. IR and chronic inflammation are suggested as possible mechanisms connecting these conditions to cardiometabolic diseases [37, 38]. In hepatic steatosis, IR manifests as decreased insulin sensitivity in the skeletal muscle [39], liver [40], and adipose tissue [41]. Additionally, steatotic liver disease triggers chronic systemic inflammation, characterized by the aberrant activation and accumulation of immune cells, which further contributes to CVD [42, 43]. However, the influence of IR and systemic inflammation on the relationship between prediabetes, hepatic steatosis, and cardiometabolic diseases could not be determined in this study, as these factors were not evaluated in the participants. Thus, further research is needed to explore whether IR and chronic inflammation mediate the relationship between prediabetes, hepatic steatosis, and cardiometabolic outcomes.

Our study has several strengths that enhance the reliability and applicability of our findings. A notable strength is the large sample size and nationwide coverage, which improve the generalizability of our results to the broader population. The longitudinal design of our study enabled robust tracking of changes over time, providing a comprehensive view of how both conditions influenced health outcomes in middle-aged adults. Moreover, we ensured rigorous risk adjustment to minimize the influence of potential confounding variables, thus strengthening the validity of the observed associations. By employing validated biomarkers and focusing specifically on middle-aged adults, our study provides precise insights that are crucial for developing early intervention strategies to mitigate the burden of cardiometabolic diseases.

Despite these strengths, our study has some limitations that warrant consideration. First, we relied solely on fasting glucose owing to the absence of data on glycated hemoglobin (HbA1c) and the 75 g oral glucose tolerance test (OGTT) data in the NHIS claims database. While guidelines recommend using HbA1c and 2-h postprandial glucose measurements from a 75 g OGTT, along with fasting glucose, for diagnosing prediabetes [44, 45], these additional measures were not available for our analysis. Second, hepatic steatosis was assessed using the FLI, which relies on surrogate markers rather than direct imaging or biopsy confirmation. While FLI is widely used and validated for predicting hepatic steatosis in large population studies, it does not distinguish between various stages of liver disease, such as steatohepatitis or fibrosis [32, 46, 47]. Nevertheless, the FLI remains a well-established, noninvasive biomarker for predicting hepatic steatosis, with validation in Asian populations and globally [17, 48]. Third, our study predominantly included Korean participants, which limits the generalizability of our findings to other ethnic groups with different genetic backgrounds and lifestyles. Fourth, our study is limited by the fact that diagnoses were made solely by the claims data, rather than through a structured follow-up protocol. Therefore, accessibility to the medical system may have influenced the detection of outcome diagnoses. However, since the NHIS covers nearly the entire Korean population and access to healthcare in Korea is generally high [49, 50], we believe this issue would have had minimal impact. Fifth, we did not assess other important cardiometabolic outcomes, such as heart failure and atherosclerotic coronary disease including stable angina and unstable angina. Our study focused on assessing hard cardiovascular outcomes, and we therefore utilized 3-point MACE as the primary endpoint, consistent with other cardiovascular outcome trials evaluating glucose-lowering medications [51–53]. However, since cardiovascular death and all-cause mortality include deaths attributable to heart failure and atherosclerotic coronary disease, individuals who died from these conditions are reflected in the outcomes of our study. Sixth, the definitions of prediabetes and FLI-based hepatic steatosis used in this study both include components of metabolic syndrome, such as impaired fasting glucose, waist circumference, and triglyceride levels [54]. This overlap makes it challenging to regard them as entirely independent entities rather than proxies for metabolic syndrome. However, as shown in Supplementary Table 1, there is a substantial degree of discordance between the two conditions, indicating that they do not completely overlap. Furthermore, findings from a previous study that defined hepatic steatosis using abdominal ultrasonography support our observation that prediabetes and hepatic steatosis do not always coexist [55]. This suggests that it is appropriate to consider these two conditions as related but distinct entities and to evaluate their independent and combined impacts on cardiometabolic outcomes. Lastly, despite our efforts to exclude patients with prior cardiovascular events to minimize reverse causality, the design of our study does not establish a causal relationship between prediabetes, hepatic steatosis, and adverse health outcomes.

## Conclusions

Our study underscores the independent and combined risks associated with prediabetes and hepatic steatosis for incident DM, composite MACE, and all-cause mortality in middle-aged adults. These findings highlight the critical importance of early detection and comprehensive management of both conditions to mitigate the burden of cardiometabolic diseases and improve long-term health outcomes. However, further research is necessary to validate these findings in diverse populations and explore their underlying mechanisms.

## Electronic supplementary material

Below is the link to the electronic supplementary material.

Supplementary Material 1

## Data Availability

Additional data can be accessed with approval and oversight from the Korean National Health Insurance Service. The data that supporting the findings of this study are publicly available at https://nhiss.nhis.or.kr/bd/ay/bdaya001iv.do. The corresponding author (WSC) confirm that this manuscript provides a honest, accurate, and transparent account of the reported study, that no significant aspects have been left out, and that any deviations from the planned study (and, if applicable, its registration) have been fully addressed.

## Abbreviations

BMI: Body mass index
BP: Blood pressure
CKD: Chronic kidney disease
CVD: Cardiovascular disease
DM: Diabetes mellitus
FLI: Fatty liver index
GGT: γ-Glutamyl transferase
HTN: Hypertension
IR: Insulin resistance
MACE: Major adverse cardiovascular events
MI: Myocardial infarction
NA: Not applicable
NHIS: Korean National Health Insurance
OGTT: Oral glucose tolerance test
ORs: Odds ratios
TG: Triglyceride
