# Supplementary material for: Combined impact of prediabetes and fatty liver index on cardiometabolic outcomes and mortality in middle aged adults: a nationwide cohort study
Source: Cardiovasc Diabetol. 2025 Jul 10;24:279. doi: 10.1186/s12933-025-02793-7 (PMC12247331; doi:10.1186/s12933-025-02793-7)

| **Supplementary Table 1.** Baseline characteristics of the study participants based on prediabetes and FLI status | | | | | | |
| --- | --- | --- | --- | --- | --- | --- |
|  |  | Normoglycemia & FLI < 60 (*n*=831,653) | Prediabetes & FLI < 60 (*n*=247,734) | Normoglycemia & FLI ≥ 60 (*n*=60,372) | Prediabetes & FLI ≥ 60 (*n*=42,992) | *P*-value |
| Age, years |  | 48.6 ± 6.7 | 49.9 ± 6.9 | 48.4 ± 6.7 | 49.0 ± 6.7 | <0.001 |
| Male |  | 290,100 (34.9) | 112,870 (45.6) | 47,457 (78.6) | 34,272 (79.7) | <0.001 |
| Income level, lowest 25% |  | 173,924 (20.9) | 51,077 (20.6) | 10,689 (17.7) | 7,514 (17.5) | <0.001 |
| Smoking |  |  |  |  |  | <0.001 |
| Nonsmoker |  | 612,102 (73.6) | 165,959 (67.0) | 24,538 (40.6) | 17,204 (40.0) |  |
| Former smoker |  | 84,606 (10.2) | 34,324 (13.9) | 12,539 (20.8) | 9,751 (22.7) |  |
| Current smoker |  | 134,945 (16.2) | 47,451 (19.2) | 23,295 (38.6) | 16,037 (37.3) |  |
| Alcohol |  |  |  |  |  | <0.001 |
| None |  | 550,684 (66.2) | 150,353 (60.7) | 26,191 (43.4) | 17,300 (40.2) |  |
| Mild |  | 280,969 (33.8) | 97,381 (39.3) | 34,181 (56.6) | 25,692 (59.8) |  |
| Regular physical activity |  | 148,221 (17.8) | 45,697 (18.5) | 9,367 (15.5) | 6,770 (15.8) | <0.001 |
| Body weight, kg |  | 60.1 ± 9.1 | 62.6 ± 9.3 | 77.9 ± 9.5 | 78.0 ± 9.7 | <0.001 |
| BMI |  |  |  |  |  | <0.001 |
| < 18.5 kg/m^2^ |  | 24,542 (3.0) | 4,619 (1.9) | 10 (0.0) | 14 (0.0) |  |
| 18.5-22.9 kg/m^2^ |  | 392,427 (47.2) | 92,614 (37.4) | 1,385 (2.3) | 1,018 (2.4) |  |
| 23.0-24.9 kg/m^2^ |  | 222,891 (26.8) | 73,698 (29.8) | 6,578 (10.9) | 4,640 (10.8) |  |
| 25.0-29.9 kg/m^2^ |  | 184,300 (22.2) | 73,686 (29.7) | 39,592 (65.6) | 28,204 (65.6) |  |
| ≥ 30.0 kg/m^2^ |  | 7,493 (0.9) | 3,117 (1.3) | 12,807 (21.2) | 9,116 (21.2) |  |
| Waist circumference |  |  |  |  |  |  |
| In men |  | 81.5 ± 6.4 | 82.6 ± 6.2 | 91.7 ± 6.2 | 91.9 ± 6.6 | <0.001 |
| In women |  | 74.9 ± 7.2 | 77.0 ± 7.4 | 92.3 ± 7.5 | 91.7 ± 6.3 | <0.001 |
| SBP, mmHg |  | 118.9 ± 14.3 | 123.6 ± 14.9 | 128.5 ± 14.6 | 130.5 ± 15.1 | <0.001 |
| DBP, mmHg |  | 74.5 ± 9.9 | 77.3 ± 10.1 | 81.3 ± 10.3 | 82.5 ± 10.5 | <0.001 |
| Fasting glucose, mg/dL |  | 88.1 ± 7.3 | 107.1 ± 6.2 | 89.6 ± 7.3 | 109.0 ± 6.9 | <0.001 |
| Total cholesterol, mg/dL |  | 196.4 ± 37.6 | 204.3 ± 41.1 | 214.4 ± 38.8 | 218.2 ± 45.8 | <0.001 |
| Triglycerides, mg/dL |  | 109.1 ± 63.0 | 123.7 ± 67.7 | 255.7 ± 172.7 | 257.9 ± 159.2 | <0.001 |
| HDL-C, mg/dL |  | 57.4 ± 25.8 | 56.4 ± 31.6 | 51.5 ± 47.6 | 50.2 ± 33.4 | <0.001 |
| LDL-C, mg/dL |  | 119.4 ± 63.1 | 125.0 ± 66.2 | 122.6 ± 105.5 | 122.1 ± 92.8 | <0.001 |
| AST, IU/L^a^ |  | 21 (18-25) | 22 (19-27) | 27 (22-35) | 28 (23-36) | <0.001 |
| ALT, IU/L^a^ |  | 18 (14-24) | 20 (15-27) | 33 (24-47) | 35 (26-51) | <0.001 |
| GGT, IU/L^a^ |  | 18 (13-27) | 22 (16-33) | 54 (36-85) | 60 (40-96) | <0.001 |
| Hypertension |  | 135,314 (16.3) | 59,560 (24.0) | 20,504 (34.0) | 17,022 (39.6) | <0.001 |
| Dyslipidemia |  | 135,302 (16.3) | 54,632 (22.1) | 19,293 (32.0) | 15,207 (35.4) | <0.001 |
| Chronic kidney disease |  | 517 (0.1) | 166 (0.1) | 51 (0.1) | 28 (0.1) | 0.196 |

Continuous variables are expressed as mean ± standard deviation. Categorical data are presented as frequencies and percentages. BMI, body mass index; SBP, systolic blood pressure; DBP, diastolic blood pressure; FLI, fatty liver index; HDL-C, high-density lipoprotein cholesterol; LDL-C, low-density lipoprotein cholesterol; AST, aspartate aminotransferase; ALT, alanine aminotransferase; GGT, gamma-glutamyl transferase. ^a^As the variables did not follow a normal distribution, they are presented as median (interquartile range), and group comparisons were conducted using the Kruskal-Wallis H test.

| **Supplementary Table 2.** Incidence rates and risk of outcomes based on prediabetes and FLI in middle-aged adults stratified by sex | | | | | | |  |
| --- | --- | --- | --- | --- | --- | --- | --- |
|  |  | Event | Duration (person-years) | Incidence Rate^a^ | Odds Ratio (95% CI)^b^ | *P* for interaction | |
| Incident diabetes |  |  |  |  |  | <0.001 | |
| Male |  |  |  |  |  |  | |
| Normoglycemia & FLI < 60 |  | 12,964 | 3,266,492 | 3.96 |  |  | |
| Prediabetes & FLI < 60 |  | 16,119 | 1,211,762 | 13.30 | 3.56 (3.48-3.65) |  | |
| Normoglycemia & FLI ≥ 60 |  | 6,839 | 513,008 | 13.33 | 3.60 (3.49-3.71) |  | |
| Prediabetes & FLI ≥ 60 |  | 11,007 | 333,816 | 32.97 | 10.11 (9.83-10.41) |  | |
| Female |  |  |  |  |  |  | |
| Normoglycemia & FLI < 60 |  | 20,158 | 6,081,093 | 3.31 |  |  | |
| Prediabetes & FLI < 60 |  | 20,818 | 1,435,712 | 14.50 | 4.72 (4.63-4.82) |  | |
| Normoglycemia & FLI ≥ 60 |  | 2,592 | 134,598 | 19.25 | 6.49 (6.21-6.80) |  | |
| Prediabetes & FLI ≥ 60 |  | 3,853 | 77,067 | 49.99 | 20.48 (19.59-21.41) |  | |
| Composite MACE |  |  |  |  |  | 0.001 | |
| Male |  |  |  |  |  |  | |
| Normoglycemia & FLI < 60 |  | 10,995 | 3,267,549 | 3.36 |  |  | |
| Prediabetes & FLI < 60 |  | 4,794 | 1,265,103 | 3.78 | 1.13 (1.09-1.17) |  | |
| Normoglycemia & FLI ≥ 60 |  | 2,300 | 530,343 | 4.33 | 1.29 (1.23-1.35) |  | |
| Prediabetes & FLI ≥ 60 |  | 1,813 | 379,497 | 4.77 | 1.42 (1.35-1.49) |  | |
| Female |  |  |  |  |  |  | |
| Normoglycemia & FLI < 60 |  | 11,698 | 6,106,210 | 1.91 |  |  | |
| Prediabetes & FLI < 60 |  | 3,410 | 1,517,715 | 2.24 | 1.17 (1.13-1.22) |  | |
| Normoglycemia & FLI ≥ 60 |  | 522 | 143,488 | 3.63 | 1.91 (1.74-2.09) |  | |
| Prediabetes & FLI ≥ 60 |  | 376 | 96,327 | 3.90 | 2.04 (1.84-2.27) |  | |
| All-cause mortality |  |  |  |  |  | <0.001 | |
| Male |  |  |  |  |  |  | |
| Normoglycemia & FLI < 60 |  | 8,737 | 3,312,960 | 2.64 |  |  | |
| Prediabetes & FLI < 60 |  | 3,977 | 1,284,668 | 3.10 | 1.18 (1.13-1.22) |  | |
| Normoglycemia & FLI ≥ 60 |  | 1,610 | 539,859 | 2.98 | 1.13 (1.07-1.19) |  | |
| Prediabetes & FLI ≥ 60 |  | 1,368 | 387,294 | 3.53 | 1.34 (1.26-1.42) |  | |
| Female |  |  |  |  |  |  | |
| Normoglycemia & FLI < 60 |  | 6,772 | 6,153,673 | 1.10 |  |  | |
| Prediabetes & FLI < 60 |  | 2,126 | 1,531,570 | 1.39 | 1.26 (1.20-1.33) |  | |
| Normoglycemia & FLI ≥ 60 |  | 299 | 145,610 | 2.05 | 1.87 (1.66-2.10) |  | |
| Prediabetes & FLI ≥ 60 |  | 241 | 97,896 | 2.46 | 2.25 (1.97-2.56) |  | |

Abbreviation: BMI, body mass index; CI, confidence interval; FLI, fatty liver index; MACE, major adverse cardiovascular events. ^a^Incidence for 1000 person-years. ^b^Adjusted for age, sex, income, smoking status, alcohol consumption, regular physical activity, body weight, hypertension, dyslipidemia, and chronic kidney disease.

| **Supplementary Table 3.** Incidence rates and odds ratios for outcomes based on prediabetes and FLI in middle-aged adults stratified by income level (lowest 25% vs. rest) | | | | | | |  |
| --- | --- | --- | --- | --- | --- | --- | --- |
|  |  | Event | Duration (person-years) | Incidence Rate^a^ | Odds Ratio (95% CI)^b^ | *P* for interaction | |
| Incident diabetes |  |  |  |  |  | <0.001 | |
| Lowest 25% income |  |  |  |  |  |  | |
| Normoglycemia & FLI < 60 |  | 7,899 | 1,945,579 | 4.05 |  |  | |
| Prediabetes & FLI < 60 |  | 8,144 | 538,883 | 15.11 | 3.99 (3.86-4.12) |  | |
| Normoglycemia & FLI ≥ 60 |  | 1,970 | 111,243 | 17.70 | 4.75 (4.50-5.01) |  | |
| Prediabetes & FLI ≥ 60 |  | 2,768 | 69,676 | 39.72 | 12.26 (11.64-12.91) |  | |
| Rest |  |  |  |  |  |  | |
| Normoglycemia & FLI < 60 |  | 25,223 | 7,402,006 | 3.40 |  |  | |
| Prediabetes & FLI < 60 |  | 28,793 | 2,108,590 | 13.65 | 4.30 (4.23-4.38) |  | |
| Normoglycemia & FLI ≥ 60 |  | 7,461 | 536,363 | 13.91 | 4.43 (4.31-4.56) |  | |
| Prediabetes & FLI ≥ 60 |  | 12,092 | 341,208 | 35.43 | 12.97 (12.64-13.3) |  | |
| Composite MACE |  |  |  |  |  | <0.001 | |
| Lowest 25% income |  |  |  |  |  |  | |
| Normoglycemia & FLI < 60 |  | 5,196 | 1,953,570 | 2.65 |  |  | |
| Prediabetes & FLI < 60 |  | 1,857 | 569,012 | 3.26 | 1.23 (1.16-1.29) |  | |
| Normoglycemia & FLI ≥ 60 |  | 613 | 117,205 | 5.23 | 1.98 (1.81-2.15) |  | |
| Prediabetes & FLI ≥ 60 |  | 473 | 81,539 | 5.80 | 2.18 (1.98-2.40) |  | |
| Rest |  |  |  |  |  |  | |
| Normoglycemia & FLI < 60 |  | 17,497 | 7,420,189 | 2.35 |  |  | |
| Prediabetes & FLI < 60 |  | 6,347 | 2,213,806 | 2.86 | 1.22 (1.19-1.26) |  | |
| Normoglycemia & FLI ≥ 60 |  | 2,209 | 556,626 | 3.96 | 1.70 (1.63-1.78) |  | |
| Prediabetes & FLI ≥ 60 |  | 1,716 | 394,285 | 4.35 | 1.86 (1.77-1.96) |  | |
| All-cause mortality |  |  |  |  |  | <0.001 | |
| Lowest 25% income |  |  |  |  |  |  | |
| Normoglycemia & FLI < 60 |  | 3,852 | 1,974,352 | 1.95 |  |  | |
| Prediabetes & FLI < 60 |  | 1,619 | 576,418 | 2.81 | 1.45 (1.36-1.53) |  | |
| Normoglycemia & FLI ≥ 60 |  | 504 | 119,573 | 4.21 | 2.19 (1.99-2.40) |  | |
| Prediabetes & FLI ≥ 60 |  | 360 | 83,546 | 4.31 | 2.22 (1.99-2.48) |  | |
| Income else |  |  |  |  |  |  | |
| Normoglycemia & FLI < 60 |  | 11,657 | 7,492,281 | 1.56 |  |  | |
| Prediabetes & FLI < 60 |  | 4,484 | 2,239,821 | 2.00 | 1.29 (1.25-1.34) |  | |
| Normoglycemia & FLI ≥ 60 |  | 1,405 | 565,896 | 2.48 | 1.61 (1.52-1.71) |  | |
| Prediabetes & FLI ≥ 60 |  | 1,249 | 401,645 | 3.11 | 2.02 (1.91-2.15) |  | |

Abbreviation: BMI, body mass index; CI, confidence interval; FLI, fatty liver index; MACE, major adverse cardiovascular events. ^a^Incidence for 1000 person-years. ^b^Adjusted for age, sex, income, smoking status, alcohol consumption, regular physical activity, body weight, hypertension, dyslipidemia, and chronic kidney disease.

| **Supplementary Table 4.** Incidence rates and odds ratios for outcomes based on prediabetes and FLI in middle-aged adults stratified by smoking status | | | | | | |  |
| --- | --- | --- | --- | --- | --- | --- | --- |
|  |  | Event | Duration (person-years) | Incidence Rate^a^ | Odds Ratio (95% CI)^b^ | *P* for interaction | |
| Incident diabetes |  |  |  |  |  | <0.001 | |
| Non-current smoker |  |  |  |  |  |  | |
| Normoglycemia & FLI < 60 |  | 25,671 | 7,851,940 | 3.26 |  |  | |
| Prediabetes & FLI < 60 |  | 29,081 | 2,149,801 | 13.52 | 4.44 (4.36-4.52) |  | |
| Normoglycemia & FLI ≥ 60 |  | 5,592 | 399,372 | 14.00 | 4.64 (4.50-4.79) |  | |
| Prediabetes & FLI ≥ 60 |  | 9,213 | 258,511 | 35.63 | 13.57 (13.2-13.96) |  | |
| Current smoker |  |  |  |  |  |  | |
| Normoglycemia & FLI < 60 |  | 7,451 | 1,495,645 | 4.98 |  |  | |
| Prediabetes & FLI < 60 |  | 7,856 | 497,673 | 15.78 | 3.39 (3.28-3.51) |  | |
| Normoglycemia & FLI ≥ 60 |  | 3,839 | 248,234 | 15.46 | 3.38 (3.24-3.52) |  | |
| Prediabetes & FLI ≥ 60 |  | 5,647 | 152,372 | 37.06 | 9.30 (8.93-9.68) |  | |
| Composite MACE |  |  |  |  |  | <0.001 | |
| Non-current smoker |  |  |  |  |  |  | |
| Normoglycemia & FLI < 60 |  | 16,521 | 7,877,453 | 2.09 |  |  | |
| Prediabetes & FLI < 60 |  | 5,677 | 2,260,337 | 2.51 | 1.20 (1.16-1.24) |  | |
| Normoglycemia & FLI ≥ 60 |  | 1,449 | 416,354 | 3.48 | 1.67 (1.59-1.77) |  | |
| Prediabetes & FLI ≥ 60 |  | 1,165 | 300,507 | 3.87 | 1.86 (1.75-1.98) |  | |
| Current smoker |  |  |  |  |  |  | |
| Normoglycemia & FLI < 60 |  | 6,172 | 1,496,306 | 4.12 |  |  | |
| Prediabetes & FLI < 60 |  | 2,527 | 522,481 | 4.83 | 1.17 (1.12-1.23) |  | |
| Normoglycemia & FLI ≥ 60 |  | 1,373 | 257,477 | 5.33 | 1.31 (1.23-1.39) |  | |
| Prediabetes & FLI ≥ 60 |  | 1,024 | 175,317 | 5.84 | 1.42 (1.33-1.52) |  | |
| All-cause mortality |  |  |  |  |  | <0.001 | |
| Non-current smoker |  |  |  |  |  |  | |
| Normoglycemia & FLI < 60 |  | 10,533 | 7,944,673 | 1.33 |  |  | |
| Prediabetes & FLI < 60 |  | 4,020 | 2,283,270 | 1.76 | 1.33 (1.29-1.38) |  | |
| Normoglycemia & FLI ≥ 60 |  | 1,019 | 422,261 | 2.41 | 1.84 (1.72-1.97) |  | |
| Prediabetes & FLI ≥ 60 |  | 838 | 305,483 | 2.74 | 2.09 (1.95-2.25) |  | |
| Current smoker |  |  |  |  |  |  | |
| Normoglycemia & FLI < 60 |  | 4,976 | 1,521,960 | 3.27 |  |  | |
| Prediabetes & FLI < 60 |  | 2,083 | 532,969 | 3.91 | 1.20 (1.14-1.26) |  | |
| Normoglycemia & FLI ≥ 60 |  | 890 | 263,208 | 3.38 | 1.04 (0.96-1.12) |  | |
| Prediabetes & FLI ≥ 60 |  | 771 | 179,708 | 4.29 | 1.32 (1.22-1.43) |  | |

Abbreviation: BMI, body mass index; CI, confidence interval; FLI, fatty liver index; MACE, major adverse cardiovascular events. ^a^Incidence for 1000 person-years. ^b^Adjusted for age, sex, income, smoking status, alcohol consumption, regular physical activity, body weight, hypertension, dyslipidemia, and chronic kidney disease.

| **Supplementary Table 5.** Incidence rates and odds ratios for outcomes based on prediabetes and FLI in middle-aged adults stratified by alcohol consumption history | | | | | | |  |
| --- | --- | --- | --- | --- | --- | --- | --- |
|  |  | Event | Duration (person-years) | Incidence Rate^a^ | Odds Ratio (95% CI)^b^ | *P* for interaction | |
| Incident diabetes |  |  |  |  |  | <0.001 | |
| Non-alcohol consumption |  |  |  |  |  |  | |
| Normoglycemia & FLI < 60 |  | 23,118 | 6,178,873 | 3.74 |  |  | |
| Prediabetes & FLI < 60 |  | 24,011 | 1,596,514 | 15.03 | 4.34 (4.26-4.42) |  | |
| Normoglycemia & FLI ≥ 60 |  | 4,780 | 276,414 | 17.29 | 5.09 (4.92-5.27) |  | |
| Prediabetes & FLI ≥ 60 |  | 6,776 | 158,992 | 42.61 | 14.69 (14.21-15.19) |  | |
| Mild alcohol consumption |  |  |  |  |  |  | |
| Normoglycemia & FLI < 60 |  | 10,004 | 3,168,713 | 3.15 |  |  | |
| Prediabetes & FLI < 60 |  | 12,926 | 1,050,960 | 12.29 | 4.15 (4.03-4.26) |  | |
| Normoglycemia & FLI ≥ 60 |  | 4,651 | 371,192 | 12.52 | 4.27 (4.11-4.43) |  | |
| Prediabetes & FLI ≥ 60 |  | 8,084 | 251,891 | 32.09 | 12.44 (12.03-12.85) |  | |
| Composite MACE |  |  |  |  |  | <0.001 | |
| Non-alcohol consumption |  |  |  |  |  |  | |
| Normoglycemia & FLI < 60 |  | 15,158 | 6,201,332 | 2.44 |  |  | |
| Prediabetes & FLI < 60 |  | 5,072 | 1,687,377 | 3.00 | 1.23 (1.19-1.27) |  | |
| Normoglycemia & FLI ≥ 60 |  | 1,376 | 290,605 | 4.73 | 1.96 (1.85-2.07) |  | |
| Prediabetes & FLI ≥ 60 |  | 946 | 190,810 | 4.95 | 2.04 (1.91-2.19) |  | |
| Mild alcohol consumption |  |  |  |  |  |  | |
| Normoglycemia & FLI < 60 |  | 7,535 | 3,172,427 | 2.37 |  |  | |
| Prediabetes & FLI < 60 |  | 3,132 | 1,095,441 | 2.85 | 1.21 (1.16-1.26) |  | |
| Normoglycemia & FLI ≥ 60 |  | 1,446 | 383,226 | 3.77 | 1.60 (1.51-1.70) |  | |
| Prediabetes & FLI ≥ 60 |  | 1,243 | 285,014 | 4.36 | 1.84 (1.74-1.96) |  | |
| All-cause mortality |  |  |  |  |  | 0.011 | |
| Non-alcohol consumption |  |  |  |  |  |  | |
| Normoglycemia & FLI < 60 |  | 10,055 | 6,263,265 | 1.61 |  |  | |
| Prediabetes & FLI < 60 |  | 3,556 | 1,708,222 | 2.08 | 1.30 (1.25-1.35) |  | |
| Normoglycemia & FLI ≥ 60 |  | 858 | 296,285 | 2.90 | 1.82 (1.70-1.95) |  | |
| Prediabetes & FLI ≥ 60 |  | 645 | 194,804 | 3.31 | 2.08 (1.92-2.26) |  | |
| Mild alcohol consumption |  |  |  |  |  |  | |
| Normoglycemia & FLI < 60 |  | 5,454 | 3,203,368 | 1.70 |  |  | |
| Prediabetes & FLI < 60 |  | 2,547 | 1,108,016 | 2.30 | 1.36 (1.29-1.42) |  | |
| Normoglycemia & FLI ≥ 60 |  | 1,051 | 389,183 | 2.70 | 1.60 (1.50-1.71) |  | |
| Prediabetes & FLI ≥ 60 |  | 964 | 290,386 | 3.32 | 1.97 (1.84-2.11) |  | |

Abbreviation: BMI, body mass index; CI, confidence interval; FLI, fatty liver index; MACE, major adverse cardiovascular events. ^a^Incidence for 1000 person-years. ^b^Adjusted for age, sex, income, smoking status, alcohol consumption, regular physical activity, body weight, hypertension, dyslipidemia, and chronic kidney disease.

| **Supplementary Table 6.** Incidence rates and odds ratios for outcomes based on prediabetes and FLI in middle-aged adults strafitied by BMI status | | | | | | |  |
| --- | --- | --- | --- | --- | --- | --- | --- |
|  |  | Event | Duration (person-years) | Incidence Rate^a^ | Odds Ratio (95% CI)^b^ | *P* for interaction | |
| Incident diabetes |  |  |  |  |  | <0.001 | |
| BMI < 25 kg/m^2^ |  |  |  |  |  |  | |
| Normoglycemia & FLI < 60 |  | 19,085 | 7,211,953 | 2.64 |  |  | |
| Prediabetes & FLI < 60 |  | 20,267 | 1,851,452 | 10.94 | 4.38 (4.29-4.47) |  | |
| Normoglycemia & FLI ≥ 60 |  | 919 | 87,133 | 10.54 | 4.24 (3.95-4.55) |  | |
| Prediabetes & FLI ≥ 60 |  | 1,561 | 56,386 | 27.68 | 12.36 (11.64-13.13) |  | |
| BMI ≥ 25 kg/m^2^ |  |  |  |  |  |  | |
| Normoglycemia & FLI < 60 |  | 14,037 | 2,135,633 | 6.57 |  |  | |
| Prediabetes & FLI < 60 |  | 16,670 | 796,021 | 20.94 | 3.51 (3.43-3.60) |  | |
| Normoglycemia & FLI ≥ 60 |  | 8,512 | 560,473 | 15.18 | 2.46 (2.39-2.53) |  | |
| Prediabetes & FLI ≥ 60 |  | 13,299 | 354,498 | 37.51 | 7.01 (6.82-7.20) |  | |
| Composite MACE |  |  |  |  |  | <0.001 | |
| BMI < 25 kg/m^2^ |  |  |  |  |  |  | |
| Normoglycemia & FLI < 60 |  | 16,175 | 7,214,198 | 2.24 |  |  | |
| Prediabetes & FLI < 60 |  | 5,359 | 1,921,729 | 2.78 | 1.25 (1.21-1.29) |  | |
| Normoglycemia & FLI ≥ 60 |  | 422 | 89,013 | 4.74 | 2.16 (1.95-2.38) |  | |
| Prediabetes & FLI ≥ 60 |  | 271 | 62,885 | 4.30 | 1.93 (1.71-2.19) |  | |
| BMI ≥ 25 kg/m^2^ |  |  |  |  |  |  | |
| Normoglycemia & FLI < 60 |  | 6,518 | 2,159,561 | 3.01 |  |  | |
| Prediabetes & FLI < 60 |  | 2,845 | 861,089 | 3.30 | 1.09 (1.05-1.14) |  | |
| Normoglycemia & FLI ≥ 60 |  | 2,400 | 584,818 | 4.10 | 1.36 (1.30-1.43) |  | |
| Prediabetes & FLI ≥ 60 |  | 1,918 | 412,939 | 4.64 | 1.54 (1.46-1.62) |  | |
| All-cause mortality |  |  |  |  |  | <0.001 | |
| BMI < 25 kg/m^2^ |  |  |  |  |  |  | |
| Normoglycemia & FLI < 60 |  | 11,827 | 7,279,557 | 1.62 |  |  | |
| Prediabetes & FLI < 60 |  | 4,385 | 1,943,046 | 2.26 | 1.40 (1.35-1.45) |  | |
| Normoglycemia & FLI ≥ 60 |  | 400 | 90,647 | 4.41 | 2.80 (2.53-3.11) |  | |
| Prediabetes & FLI ≥ 60 |  | 312 | 63,935 | 4.88 | 3.09 (2.75-3.47) |  | |
| BMI ≥ 25 kg/m^2^ |  |  |  |  |  |  | |
| Normoglycemia & FLI < 60 |  | 3,682 | 2,187,076 | 1.68 |  |  | |
| Prediabetes & FLI < 60 |  | 1,718 | 873,193 | 1.97 | 1.17 (1.10-1.24) |  | |
| Normoglycemia & FLI ≥ 60 |  | 1,509 | 594,821 | 2.54 | 1.51 (1.43-1.61) |  | |
| Prediabetes & FLI ≥ 60 |  | 1,297 | 421,256 | 3.08 | 1.84 (1.72-1.96) |  | |

Abbreviation: BMI, body mass index; CI, confidence interval; FLI, fatty liver index; MACE, major adverse cardiovascular events. ^a^Incidence for 1000 person-years. ^b^Adjusted for age, sex, income, smoking status, alcohol consumption, regular physical activity, body weight, hypertension, dyslipidemia, and chronic kidney disease.

| **Supplementary Table 7.** Baseline characteristics of the study participants based on prediabetes and FLI status | | | | | | |
| --- | --- | --- | --- | --- | --- | --- |
|  |  | Normoglycemia & FLI < 30 (*n*=668,320) | Prediabetes & FLI < 30 (*n*=167,792) | Normoglycemia & FLI ≥ 30 (*n*=223,705) | Prediabetes & FLI ≥ 30 (*n*=122,934) | *P*-value |
| Age, years |  | 48.35 ± 6.65 | 49.57 ± 6.83 | 49.35 ± 6.87 | 49.92 ± 6.85 | <0.001 |
| Male |  | 184,980 (27.68) | 60,136 (35.84) | 152,577 (68.2) | 87,006 (70.77) | <0.001 |
| Income level, lowest 25% |  | 144,365 (21.6) | 36,588 (21.81) | 40,248 (17.99) | 22,003 (17.9) | <0.001 |
| Smoking |  |  |  |  |  |  |
| Nonsmoker |  | 524,958 (78.55) | 123,925 (73.86) | 111,682 (49.92) | 59,238 (48.19) | <0.001 |
| Former smoker |  | 54,831 (8.2) | 18,277 (10.89) | 42,314 (18.92) | 25,798 (20.99) |  |
| Current smoker |  | 88,531 (13.25) | 25,590 (15.25) | 69,709 (31.16) | 37,898 (30.83) |  |
| Alcohol |  |  |  |  |  |  |
| None |  | 461,804 (69.1) | 109,232 (65.1) | 115,071 (51.44) | 58,421 (47.52) |  |
| Mild |  | 206,516 (30.9) | 58,560 (34.9) | 108,634 (48.56) | 64,513 (52.48) | . |
| Regular physical activity |  | 119,949 (17.95) | 31,310 (18.66) | 37,639 (16.83) | 21,157 (17.21) | <0.001 |
| Body weight, kg |  | 57.77 ± 7.76 | 59.23 ± 7.88 | 71.84 ± 9.17 | 72.50 ± 9.47 | <0.001 |
| BMI |  | 22.43 ± 2.30 | 22.80 ± 2.28 | 26.28 ± 2.65 | 26.42 ± 2.73 | <0.001 |
| < 18.5 kg/m^2^ |  | 24451 (3.66) | 4564 (2.72) | 101 (0.05) | 69 (0.06) | <0.001 |
| 18.5-22.9 kg/m^2^ |  | 376,297 (56.3) | 84,394 (50.3) | 17,515 (7.83) | 9,238 (7.51) | . |
| 23.0-24.9 kg/m^2^ |  | 175,749 (26.3) | 50,374 (30.02) | 53,720 (24.01) | 27,964 (22.75) | . |
| 25.0-29.9 kg/m^2^ |  | 90,822 (13.59) | 28,202 (16.81) | 133,070 (59.48) | 73,688 (59.94) | . |
| ≥ 30.0 kg/m^2^ |  | 1,001 (0.15) | 258 (0.15) | 19,299 (8.63) | 11,975 (9.74) | . |
| Waist circumference |  | 75.14 ± 6.64 | 76.59 ± 6.44 | 87.32 ± 6.27 | 87.82 ± 6.48 | <0.001 |
| In men |  | 78.89 ± 5.69 | 79.48 ± 5.56 | 87.83 ± 5.91 | 88.30 ± 6.15 | <0.001 |
| In women |  | 73.70 ± 6.41 | 74.98 ± 6.34 | 86.24 ± 6.86 | 86.64 ± 7.07 | <0.001 |
| SBP, mmHg |  | 117.51 ± 13.96 | 121.87 ± 14.67 | 125.74 ± 14.39 | 128.32 ± 15.01 | <0.001 |
| DBP, mmHg |  | 73.47 ± 9.70 | 76.08 ± 9.93 | 79.22 ± 10.02 | 80.75 ± 10.32 | <0.001 |
| Fasting glucose, mg/dL |  | 87.87 ± 7.34 | 106.72 ± 6.05 | 89.27 ± 7.28 | 108.30 ± 6.67 | <0.001 |
| Total cholesterol, mg/dL |  | 193.76 ± 36.88 | 200.91 ± 40.57 | 209.08 ± 38.57 | 213.72 ± 43.01 | <0.001 |
| Triglycerides, mg/dL |  | 94.35 ± 45.73 | 102.01 ± 47.27 | 192.72 ± 121.33 | 200.27 ± 121.81 | <0.001 |
| HDL-C, mg/dL |  | 58.61 ± 22.71 | 58.46 ± 33.33 | 51.98 ± 38.92 | 51.30 ± 29.35 | <0.001 |
| LDL-C, mg/dL |  | 117.86 ± 61.63 | 123.64 ± 67.73 | 124.96 ± 80.26 | 125.84 ± 74.79 | <0.001 |
| AST, IU/L^a^ |  | 21 (18-25) | 21 (18-25) | 25 (20-30) | 25 (21-32) | <0.001 |
| ALT, IU/L^a^ |  | 16 (13-22) | 18 (14-23) | 27 (20-37) | 28 (21-40) | <0.001 |
| GGT, IU/L^a^ |  | 17 (13-23) | 18 (14-25) | 36 (25-56) | 41 (28-65) | <0.001 |
| Hypertension |  | 92,251 (13.8) | 34,060 (20.3) | 63,567 (28.42) | 42,522 (34.59) | <0.001 |
| Dyslipidemia |  | 94,369 (14.12) | 31,951 (19.04) | 60,226 (26.92) | 37,888 (30.82) | <0.001 |
| Chronic kidney disease |  | 406 (0.06) | 115 (0.07) | 162 (0.07) | 79 (0.06) | 0.254 |

Continuous variables are expressed as mean ± standard deviation. Categorical data are presented as frequencies and percentages. ALT, alanine aminotransferase; AST, aspartate aminotransferase; BMI, body mass index; DBP, diastolic blood pressure; FLI, fatty liver index; GGT, gamma-glutamyl transferase; HDL-C, high-density lipoprotein cholesterol; LDL-C, low-density lipoprotein cholesterol; SBP, systolic blood pressure. ^a^As the variables did not follow a normal distribution, they are presented as median (interquartile range), and group comparisons were conducted using the Kruskal-Wallis H test.

| **Supplementary Table 8.** Incidence rates and risk of outcomes based on prediabetes and FLI in middle-aged adults (FLI < 30 vs. FLI ≥ 30) | | | | | | | | | | | | | |  |
| --- | --- | --- | --- | --- | --- | --- | --- | --- | --- | --- | --- | --- | --- | --- |
|  |  | Event | Duration (person-years) | Incidence Rate^a^ | Hazard Ratio | | | | | Odds ratio | | | |  |
|  |  |  |  |  | Model 1  (95% CI) | *P*-value | Model 2  (95% CI) | *P*-value | Model 1  (95% CI) | | *P*-value | Model 2  (95% CI) | *P*-value |  |
| Incident diabetes |  |  |  |  |  |  |  |  |  | |  |  |  |  |
| Normoglycemia & FLI < 30 |  | 18,824 | 7,537,105 | 2.50 | NA | NA | NA | NA | Reference | |  | Reference |  |  |
| Prediabetes & FLI < 30 |  | 18,175 | 1,828,332 | 9.94 |  |  |  |  | 4.10  (4.01-4.18) | | <0.001 | 3.90  (3.82-3.98) | <0.001 |  |
| Normoglycemia & FLI ≥ 30 |  | 23,729 | 2,458,087 | 9.65 |  |  |  |  | 4.32  (4.23-4.41) | | <0.001 | 2.83  (2.77-2.9) | <0.001 |  |
| Prediabetes & FLI ≥ 30 |  | 33,622 | 1,230,026 | 27.33 |  |  |  |  | 13.72  (13.44-14.00) | | <0.001 | 8.88  (8.68-9.09) | <0.001 |  |
| *P*_trend_ |  |  |  |  |  |  |  |  |  | | <0.001 |  | <0.001 |  |
| Composite MACE |  |  |  |  |  |  |  |  |  | |  |  |  |  |
| Normoglycemia & FLI < 30 |  | 15,940 | 7,538,647 | 2.11 | NA | NA | NA | NA | Reference | |  | Reference |  |  |
| Prediabetes & FLI < 30 |  | 4,638 | 1,889,879 | 2.45 |  |  |  |  | 1.02  (0.99-1.06) | | <0.001 | 1.00  (0.97-1.03) | 0.994 |  |
| Normoglycemia & FLI ≥ 30 |  | 9,575 | 2,508,943 | 3.82 |  |  |  |  | 1.44  (1.40-1.48) | | <0.001 | 1.32  (1.28-1.37) | <0.001 |  |
| Prediabetes & FLI ≥ 30 |  | 5,755 | 1,368,763 | 4.20 |  |  |  |  | 1.50  (1.45-1.55) | | <0.001 | 1.36  (1.32-1.41) | <0.001 |  |
| *P*_trend_ |  |  |  |  |  |  |  |  |  | | <0.001 |  | <0.001 |  |
| All-cause mortality |  |  |  |  |  |  |  |  |  | |  |  |  |  |
| Normoglycemia & FLI < 30 |  | 11,291 | 7,602,823 | 1.49 | NA | NA | NA | NA | Reference | |  | Reference |  |  |
| Prediabetes & FLI < 30 |  | 3,705 | 1,907,977 | 1.94 |  |  |  |  | 1.10  (1.06-1.14) | | 0.221 | 1.11  (1.07-1.15) | <0.001 |  |
| Normoglycemia & FLI ≥ 30 |  | 6,127 | 2,549,279 | 2.40 |  |  |  |  | 1.13  (1.09-1.16) | | 0.463 | 1.28  (1.24-1.33) | <0.001 |  |
| Prediabetes & FLI ≥ 30 |  | 4,007 | 1,393,453 | 2.88 |  |  |  |  | 1.26  (1.21-1.31) | | <0.001 | 1.45  (1.39-1.51) | <0.001 |  |
| *P*_trend_ |  |  |  |  |  |  |  |  |  | | <0.001 |  | <0.001 |  |
| Abbreviation: CI, confidence interval; FLI, fatty liver index, MACE, major adverse cardiovascular events. ^a^Incidence for 1000 person-years. Model 1: Adjusted for age and sex. Model 2: Adjusted for age, sex, income, smoking status, alcohol consumption, regular physical activity, body weight, hypertension, dyslipidemia, and chronic kidney disease. | | | | | | | | | | | | | | |

| **Supplementary Table 9.** Incidence rates and risk of outcomes based on prediabetes and FLI in middle-aged adults (5-year outcome) | | | | | | | | | | | | | |  |
| --- | --- | --- | --- | --- | --- | --- | --- | --- | --- | --- | --- | --- | --- | --- |
|  |  | Event | Duration (person-years) | Incidence Rate^a^ | Hazard Ratio | | | | Odds ratio | | | | | |
|  |  |  |  |  | Model 1  (95% CI) | *P*-value | Model 2  (95% CI) | *P*-value | | Model 1  (95% CI) | *P*-value | Model 2  (95% CI) | *P*-value | |
| Incident diabetes |  |  |  |  |  |  |  |  | |  |  |  |  | |
| Normoglycemia & FLI < 60 |  | 6,342 | 4,135,558 | 1.53 | NA | NA | NA | NA | | Reference |  | Reference |  | |
| Prediabetes & FLI < 60 |  | 10,872 | 1,211,757 | 8.97 |  |  |  |  | | 5.62  (5.45-5.80) | <0.001 | 5.19  (5.03-5.36) | <0.001 | |
| Normoglycemia & FLI ≥ 60 |  | 2,048 | 296,690 | 6.90 |  |  |  |  | | 4.82  (4.57-5.07) | 0.257 | 2.74  (2.59-2.90) | <0.001 | |
| Prediabetes & FLI ≥ 60 |  | 5,154 | 202,880 | 25.40 |  |  |  |  | | 18.31  (17.59-19.06) | <0.001 | 10.42  (9.95-10.91) | <0.001 | |
| *P*_trend_ |  |  |  |  |  |  |  |  | | <0.001 |  | <0.001 |  | |
| Composite MACE |  |  |  |  |  |  |  |  | |  |  |  |  | |
| Normoglycemia & FLI < 60 |  | 6,530 | 4,134,199 | 1.58 | NA | NA | NA | NA | | Reference |  | Reference |  | |
| Prediabetes & FLI < 60 |  | 2,410 | 1,229,366 | 1.96 |  |  |  |  | | 1.05  (1.00-1.10) | <0.001 | 1.01  (0.96-1.05) | 0.834 | |
| Normoglycemia & FLI ≥ 60 |  | 881 | 298,664 | 2.95 |  |  |  |  | | 1.50  (1.40-1.61) | <0.001 | 1.23  (1.14-1.33) | <0.001 | |
| Prediabetes & FLI ≥ 60 |  | 720 | 212,418 | 3.39 |  |  |  |  | | 1.62  (1.50-1.76) | <0.001 | 1.32  (1.21-1.43) | <0.001 | |
| *P*_trend_ |  |  |  |  |  |  |  |  | | <0.001 |  | <0.001 |  | |
| All-cause mortality |  |  |  |  |  |  |  |  | |  |  |  |  | |
| Normoglycemia & FLI < 60 |  | 4,883 | 4,147,410 | 1.18 | NA | NA | NA | NA | | Reference |  | Reference |  | |
| Prediabetes & FLI < 60 |  | 1,949 | 1,234,276 | 1.58 |  |  |  |  | | 1.11  (1.05-1.17) | 0.029 | 1.14  (1.08-1.20) | <0.001 | |
| Normoglycemia & FLI ≥ 60 |  | 574 | 300,498 | 1.91 |  |  |  |  | | 1.21  (1.10-1.32) | 0.371 | 1.48  (1.34-1.63) | <0.001 | |
| Prediabetes & FLI ≥ 60 |  | 502 | 213,877 | 2.35 |  |  |  |  | | 1.40  (1.27-1.53) | <0.001 | 1.72  (1.56-1.90) | <0.001 | |
| *P*_trend_ |  |  |  |  |  |  |  |  | | <0.001 |  | <0.001 |  | |

Abbreviation: CI, confidence interval; FLI, fatty liver index; MACE, major adverse cardiovascular events. ^a^Incidence for 1000 person-years. Model 1: Adjusted for age and sex. Model 2: Adjusted for age, sex, income, smoking status, alcohol consumption, regular physical activity, body weight, hypertension, dyslipidemia, and chronic kidney disease.

| **Supplementary Table 10.** Incidence rates and hazard/odds ratios for outcomes based on prediabetes and FLI in middle-aged adults (10-year outcome) | | | | | | | | | | | | | | |
| --- | --- | --- | --- | --- | --- | --- | --- | --- | --- | --- | --- | --- | --- | --- |
|  |  | Event | Duration (person-years) | Incidence Rate^a^ | Hazard Ratio | | | | Odds ratio | | | | | |
|  |  |  |  |  | Model 1  (95% CI) | *P*-value | Model 2  (95% CI) | *P*-value | | Model 1  (95% CI) | *P*-value | Model 2  (95% CI) | *P*-value |  |
| Incident diabetes |  |  |  |  |  |  |  |  | |  |  |  |  |  |
| Normoglycemia & FLI < 60 |  | 23,733 | 8,135,266 | 2.92 | NA | NA | NA | NA | | Reference |  | Reference |  |  |
| Prediabetes & FLI < 60 |  | 29,639 | 2,323,470 | 12.76 |  |  |  |  | | 4.42  (4.35-4.50) | <0.001 | 4.09  (4.02-4.17) | <0.001 |  |
| Normoglycemia & FLI ≥ 60 |  | 7,250 | 568,697 | 12.75 |  |  |  |  | | 4.82  (4.68-4.96) | <0.001 | 2.48  (2.41-2.56) | <0.001 |  |
| Prediabetes & FLI ≥ 60 |  | 12,586 | 368,124 | 34.19 |  |  |  |  | | 14.47  (14.10-14.84) | <0.001 | 7.61  (7.39-7.83) | <0.001 |  |
| *P*_trend_ |  |  |  |  |  |  |  |  | | <0.001 |  | <0.001 |  |  |
| Composite MACE |  |  |  |  |  |  |  |  | |  |  |  |  |  |
| Normoglycemia & FLI < 60 |  | 18,110 | 8,146,416 | 2.22 | NA | NA | NA | NA | | Reference |  | Reference |  |  |
| Prediabetes & FLI < 60 |  | 6,583 | 2,417,082 | 2.72 |  |  |  |  | | 1.06  (1.03-1.09) | <0.001 | 1.02  (0.99-1.05) | 0.128 |  |
| Normoglycemia & FLI ≥ 60 |  | 2,262 | 585,312 | 3.86 |  |  |  |  | | 1.45  (1.38-1.51) | <0.001 | 1.21  (1.15-1.27) | <0.001 |  |
| Prediabetes & FLI ≥ 60 |  | 1,814 | 414,824 | 4.37 |  |  |  |  | | 1.55  (1.48-1.63) | <0.001 | 1.29  (1.22-1.36) | <0.001 |  |
| *P*_trend_ |  |  |  |  |  |  |  |  | | <0.001 |  | <0.001 |  |  |
| All-cause mortality |  |  |  |  |  |  |  |  | |  |  |  |  |  |
| Normoglycemia & FLI < 60 |  | 12,371 | 8,210,348 | 1.51 | NA | NA | NA | NA | | Reference |  | Reference |  |  |
| Prediabetes & FLI < 60 |  | 4,909 | 2,440,347 | 2.01 |  |  |  |  | | 1.11  (1.07-1.15) | <0.001 | 1.13  (1.09-1.17) | <0.001 |  |
| Normoglycemia & FLI ≥ 60 |  | 1,508 | 593,516 | 2.54 |  |  |  |  | | 1.29  (1.22-1.37) | 0.0007 | 1.50  (1.41-1.59) | <0.001 |  |
| Prediabetes & FLI ≥ 60 |  | 1,283 | 421,505 | 3.04 |  |  |  |  | | 1.46  (1.37-1.55) | <0.001 | 1.70  (1.59-1.81) | <0.001 |  |
| *P*_trend_ |  |  |  |  |  |  |  |  | | <0.001 |  | <0.001 |  |  |

Abbreviation: CI, confidence interval; FLI, fatty liver index; MACE, major adverse cardiovascular events. ^a^Incidence for 1000 person-years. Model 1: Adjusted for age and sex. Model 2: Adjusted for age, sex, income, smoking status, alcohol consumption, regular physical activity, body weight, hypertension, dyslipidemia, and chronic kidney disease.

**Supplementary Figure 1.** Study flowchart

**
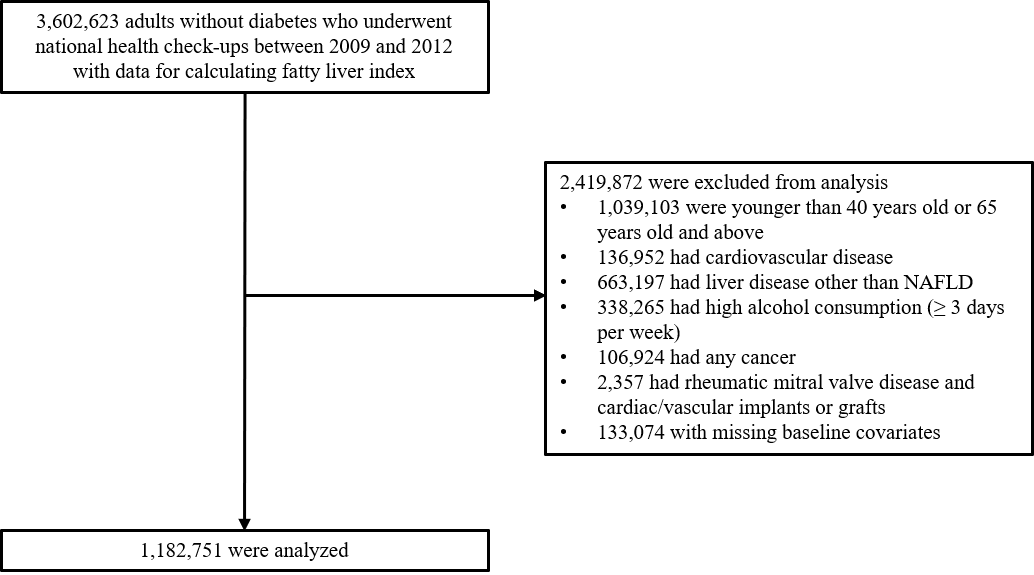
**

**Supplementary Figure 2.** Kaplan-Meier estimates of outcomes based on prediabetes status in middle-aged adults. **A.** Incident diabetes, **B.** Composite major adverse cardiovascular events, **C.** All-cause mortality


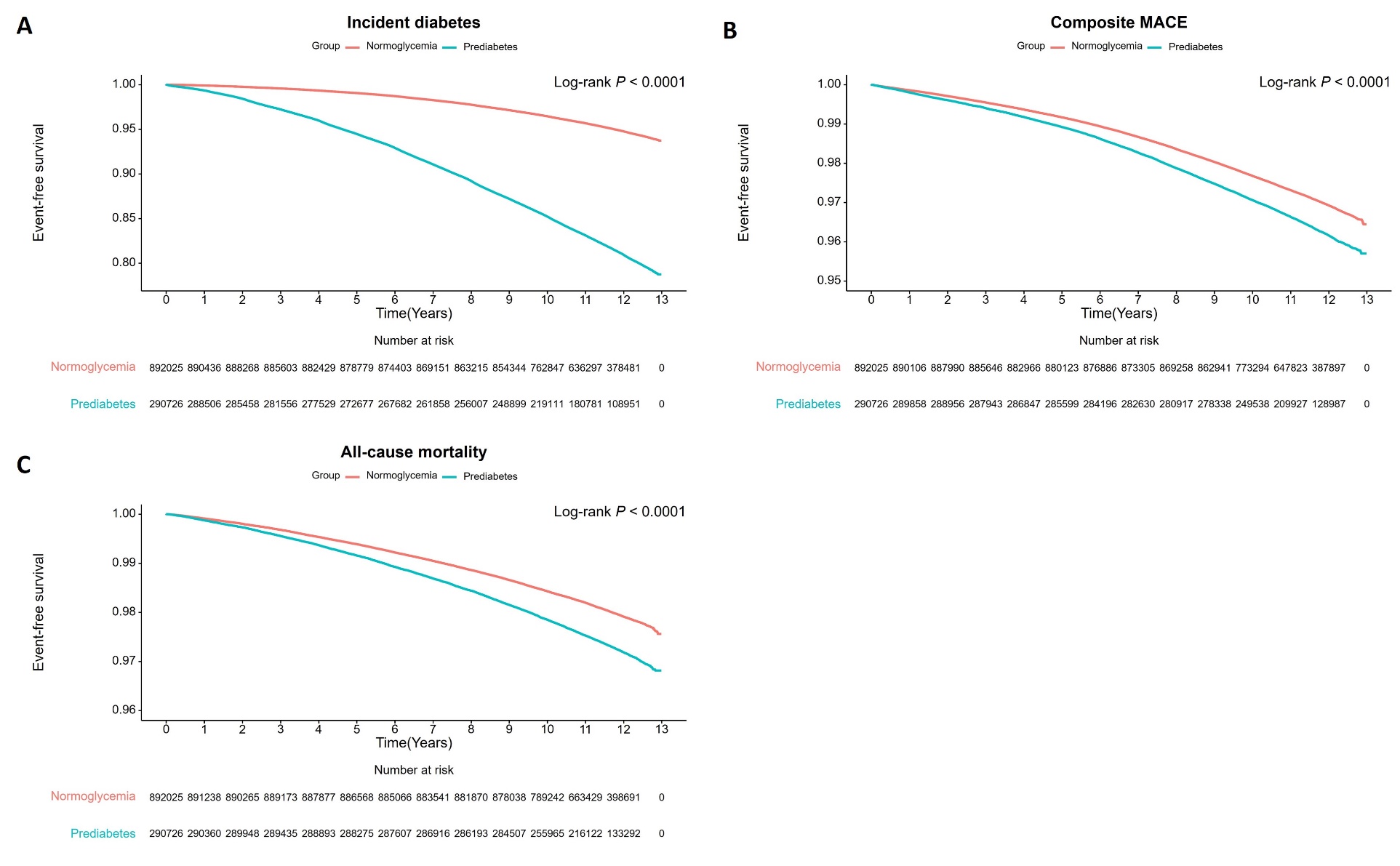


**Supplementary Figure 3.** Kaplan-Meier estimates of outcomes based on FLI status in middle-aged adults. **A.** Incident diabetes, **B.** Composite major adverse cardiovascular events, **C.** All-cause mortality


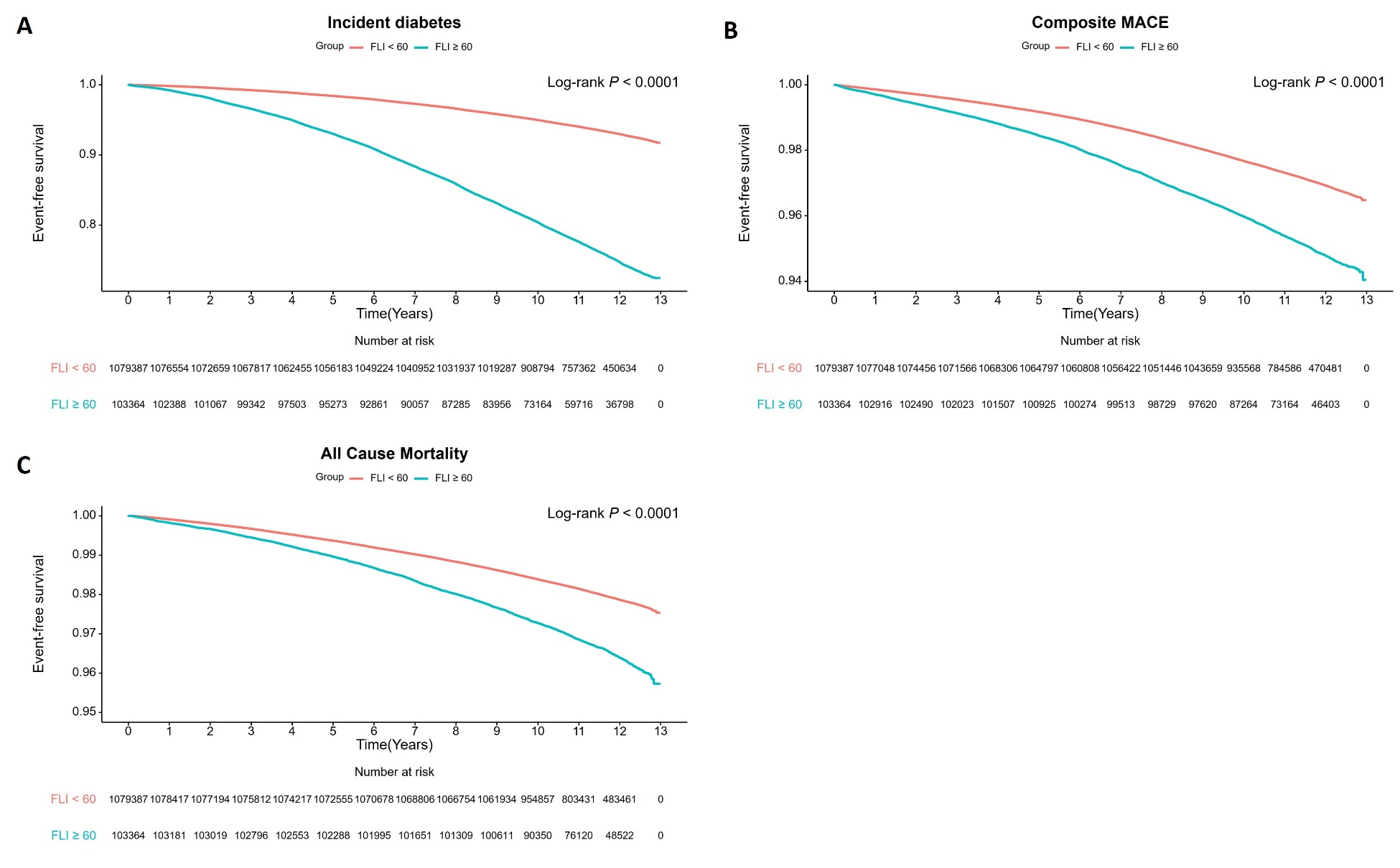

Supplement: Supplementary file 1 — Supplementary Material 1 [file 12933_2025_2793_MOESM1_ESM.docx]
